# Effect of *Centella asiatica* on pathophysiology of mild chronic cerebral hypoperfusion in rats

**Published:** 2018

**Authors:** Wachiryah Thong-asa, Kanokwan Tilokskulchai, Supin Chompoopong, Mayuree Hantrakul Tantisira

**Affiliations:** 1 *Animal Toxicology and Physiology Specialty Research Unit (ATPSRU), Physiology Division, Department of Zoology, Faculty of Science, Kasetsart University, Bangkok, Thailand*; 2 *Neuroscience Unit, Department of Physiology, Faculty of Medicine Siriraj, Siriraj Hospital, Mahidol University, Bangkok, Thailand*; 3 *Department of Anatomy, Faculty of Medicine Siriraj, Siriraj Hospital, Mahidol University, Bangkok, Thailand*; 4 *Faculty of Pharmaceutical Sciences, Burapha University, Chonburi, Thailand*; 5 *Faculty of Pharmaceutical Sciences, Chulalongkorn University, Bangkok, Thailand*

**Keywords:** Chronic cerebral hypoperfusion, Spatial cognition, Learning flexibility, Radial arm water maze, Centella asiatica

## Abstract

**Objective:**

The present study investigated the effect of standardized *Centella asiatica* extract on cognition and hippocampal pathology of mild chronic cerebral hypoperfusion (CCH) that was induced by permanent right common carotid artery occlusion (RCO) in rats.

**Materials and Methods:**

Sixty-four male Sprague-Dawley rats were randomly divided into four groups of Sham-veh, Sham-*C. asiatica*, RCO-veh and RCO-*C. asiatica*, which were further divided into short-term and long-term CCH induction. Oral treatments with 20 mg/kg *C. asiatica* initiated 24 hours and 12 months after CCH and continued for 14 consecutive days. According to the cognition and histopathological evaluation period, the experiment was divided into 2 sets of either 2 or 12 months of CCH.

**Results:**

Results showed that 2-month CCH induced learning flexibility deficit associated with CA1 neuronal damage and internal capsule (IC) astroglia activation. Long-lasting (12 months) mild CCH induced spatial learning, memory and flexibility deficits associated with progressive dorsal hippocampal damage. Treatment with 20 mg/kg of *C. asiatica* improved learning flexibility deficit after 2 and 12 months of CCH. *C. asiatica* ameliorated neuronal damage in the dorsal hippocampus at 2 months of CCH when given 24 hours after CCH onset. Treatment with *C. asiatica* after 12 months of cerebral blood flow reduction improved memory and learning flexibility deficits and was associated with the dentate gyrus neuronal damage reduction.

**Conclusion:**

Our finding indicates the therapeutic potential of *C. asiatica* either when given immediately after ischemic insult or when administered one year after ischemic insult, in a CCH rat model.

## Introduction

At present, there is no potential therapy to cure dementia and the approved therapies can only improve the symptoms but cannot modify the course of disease. Hence, an optimum treatment should act to prevent, delay or reverse the development of dementia. A number of alternative therapies has been investigated for prevention of dementia. In traditional practices of medicine, numerous plants have been used to enhance cognitive function and to alleviate other symptoms associated with dementia (Jivad and Rabiei, 2015 ▶). Animal models of CCH were proposed and used to study the pathomechanisms of vascular dementia (VD) and subcortical ischemic vascular dementia (SIVD). In the death of the neurons in CCH rat models, it had been suggested that ATP level plays a key role (Du et al., 2016 ▶; Farkas et al., 2007 ▶). If ATP suddenly drops, necrotic cell death occurs, if ATP exist but at low level (i.e. suboptimal to cell activity), the apoptotic cell death is trigged. So, under CCH condition due to lower levels of ATP such neuronal hypofunction, dysfunction and further apoptotic death, may happen. The loss of ATP is promptly followed by the dysfunction of energy-dependent ion pumps, depolarization of the neurons, and generation of reactive oxygen species (ROS). The ROS in turn, initiate lipid peroxidation and generate lipid peroxides that are degraded to reactive aldehyde products such as malondialdehyde (MDA) (Ghorbani et al., 2015 ▶; Sadeghnia et al., 2013 ▶). In parallel with the increase in lipid peroxidation, the activities of enzymatic antioxidants such as Cu/Zn-superoxide dismutase (Cu/Zn-SOD) or the concentrations of non-enzymatic antioxidants such as glutathione (GSH) are decreased. In a CCH model such as two vessel occlusion (2VO), the increase in MDA and the decrease in GSH appeared about 10 days after 2VO induction. 2VO creates a permanent ischemic/oligemic condition which is enough serious to sustain continuous oxidative stress, which could be the reason for the persistent and progressive neuronal damage (Aytac et al., 2006 ▶; Farkas et al., 2007 ▶). Neuronal dysfunction and death contribute to cognitive decline after CCH and they had been both reported in 2VO and unilateral carotid occlusion (UCO) models. However, it has been suggested that UCO model is more pathologically relevant to VD especially SIVD than the 2VO model. Using of CCH animal models provided beneficial data for improvement of neuronal functions and help cells to survive in order to prevent or delay development of dementia. 

*Centella asiatica* L. Urban is a psychoactive medicinal plant that has been used for centuries in Ayurvedic system of medicine. *C. asiatica* have cognition-enhancing properties associated with decreased oxidative stress in the brain, increased in levels of antioxidant enzymes, and ameliorated age-related decline in cognitive function and mood disorder in healthy elderly (Gupta et al., 2003 ▶; Jayashree et al., 2003 ▶; Veerendra Kumar and Gupta, 2002 ▶, 2003; Wattanathorn et al., 2008 ▶; Zainol et al., 2003 ▶). *C. asiatica* also affects neuronal morphology and acetylcholine esterase (AChE) activity. Administration of *C. asiatica* leaves extract increased dendritic arborization of hippocampal CA3 neurons and increased AChE activity in the hippocampus in young adult mice (Gadahad et al., 2008 ▶; Mohandas Rao et al., 2006b ▶, 2009). Asiatic acid, an active triterpene of *C. asiatica* had been shown to protect cortical neurons from glutamate-induced excitotoxicity (Lee et al., 2000). These changes were associated with improvement of learning and memory in the radial arm maze (RAM) (Mohandas Rao et al., 2006a ▶, b ▶; Rao et al., 2005 ▶). The beneficial effects of *C. asiatica* could be used as a preventive therapy for dementia. Administration of *C. asiatica* prior to dementia onset, during neuronal hypofunction, and before cell death might be helpful. Along with useful animal models that have been developed to study development of dementia at preclinical stages, testing preventive properties of *C. asiatica* may provide a clue for development of new effective preventive therapies. Therefore, the present study aimed to investigate the preventive effect of *C. asiatica* on development of cognitive decline and hippocampal histopathological changes in short-term CCH as well as its effect after long-term cerebral blood flow (CBF) reduction induced by permanent right common carotid artery occlusion in rats. 

## Materials and Methods

**Experimental animals**

Animals were handled according to the Guide for the Care and Use of Laboratory Animals (NIH). All animal care and experimental protocols were approved by the Animal Ethics Committee, Faculty of Medicine Siriraj, Siriraj Hospital, Mahidol University, Bangkok, Thailand. Animals were obtained from the National Laboratory Animal Centre, Mahidol University, Salaya, Nakornprathom. They were maintained under natural light/dark cycles at 25^o^ C, and allowed to have free access to standard diet and water throughout the experimental period.

**Preparation of standardized extract of** ***C. asiatica*** **(ECa-233)**

ECa-233 was prepared by collaborators at the Faculty of Pharmaceutical Sciences, Chulalongkorn University, using the well-defined procedure of whole plant extraction (patent pending). Total triterpenoids were 85% of which 53% of madecassoside and 32% of asiaticoside. In a previous study, oral administration of ECa-233 10 and 30 mg/kg to mice significantly improved learning and memory deficit induced by bilateral common arteries occlusion in both Morris water maze (MWM) and step-down tests. In addition, 2VO-induced MDA increase was abolished by ECa-233 (Wanasuntronwong et al., 2012 ▶). Therefore, in this study oral active dose selected according the above study was administered.

**Surgical procedure**

Four-month old male Sprague Dawley rats (n=64) were used. Rats were randomly divided into main groups of sham-operated (Sham) and the right common carotid occlusion rats. These main groups were further divided into two subgroups of vehicle-treated and *C. asiatica*-treated groups i.e. Sham-veh, Sham-*C. asiatica*, RCO-veh and RCO-*C. asiatica* (n=8 in each group). Sham-veh and RCO-veh were orally administered with vehicle (0.5 % carboxymethylcellulose (CMC)). Sham-*C. asiatica* and RCO - *C. asiatica* were orally administered with *C. asiatica* extracts 20 mg/kg prepared with 0.5 % CMC. These subgroups were further divided into two sets of 2 and 12 months after CCH according to the period of cognitive test and histopathological assessment .

**Administration of** ***C. asiatica*** **(ECa-233)**

*C. asiatica* extract 20 mg/kg or vehicle was administered orally once a day for 14 consecutive days starting from either 24 hours after RCO (Set I) or 12 months after RCO (Set II). The spatial learning and memory tests were assessed in the radial arm water maze (RAWM), 2 (Set I) and 12 months (Set II) after CCH.

**Sensorimotor and cognition tests in the 8-arm radial arm water maze**

Prior to the spatial learning task, each rat received a pre-training session in order to learn all procedural aspects of the task and to assess the sensorimotor function after surgery. Spatial learning and memory assessments consisted of a 5-day acquisition phase (Day 1-5) and a 3-day reversal phase (Day 6-8) and probe trial was done after finishing each phase. The RAWM test was performed four trials a day with 30 min of intertrial interval (ITI), adapted from Shukitt-Hale et al (2004) ▶ (Shukitt-Hale et al., 2004 ▶).

The RAWM was a blue circular pool (200 cm in diameter, with 50-cm high sidewall) located in a room with a variety of distal cues. The pool was filled with water (30 cm depth), and inside the pool there was a removable six-radial arm maze structure (construct by 6 V shape structures). The arms were 80-cm long and 50-cm width, the central area was 40 cm in diameter. A platform was a clear rod glass 20 cm in diameter positioned such that its top surface was submerged 2 cm below the water surface. A video-camera was fixed on the ceiling of the test room and connected to a computer for data recording. 

**Histopathological studies**

Pathological assessment was performed after behavioral tests; rats were sacrificed by an intraperitoneal injection of a lethal dose of sodium pentobarbital (>60 mg/kg). Rats were perfused intracardially with 0.9% normal saline solution (NSS) for 10 min followed by 4% paraformaldehyde in 0.2 M phosphate buffer solution (PBS), pH 7.4 for 10 min. Brains were removed and fixed in 4% paraformaldehyde in 0.2 M PBS for 24 hr. Brains were then processed for paraffin block and sectioned transversely with 5 µm of thickness by freezing microtome. The coronal serial brain sections were collected between Bregma – 2.80 to – 3.80 mm which covered the dorsal hippocampal area (Paxinos and Watson, 2006 ▶). Brain sections were selected at Bregma – 3.14 and 5 slides (125 µm of space interval) were picked from each rat for further histochemical and immunohistochemical assessments. 

**Cresyl violet staining**

Brain sections were deparaffinized in different solutions, started with two changes of xylene, 100%, 95%, 80% and 70% alcohol for 5 min each. Then, sections were hydrated using distilled water for 5 min and stained with 1.5% cresyl violet for 10 min. Washing in 95%, two changes of 100% alcohol (I, II) and acetone for 5 min each. After that, sections were cleared in xylene and mounted. Cresyl violet-stained sections were examined under light microscope (Olympus Tg300). Photomicrographs of dorsal hippocampus were captured at magnification X200. Three areas were defined on each section of dorsal hippocampus as CA1, CA3 and dentate gyrus (DG). The number of neuron in each area of dorsal hippocampus was determined by counting neurons exhibiting a visible nucleus and diameter between 15 to 35 µm in CA1 and CA3 and 9 to 25 µm in DG. Live and dead neurons of each slide were counted by using UTHSCSA Image Tool 3.0 (Thong-asa and Tilokskulchai, 2014 ▶; Thong-asa et al., 2015 ▶).

**Glia fibrillary acidic protein immunohistochemistry**

Glial fibrillary acidic protein (GFAP) immunohistochemistry was used to stain astroglia in each area of dorsal hippocampus and white matter areas. Brain sections were deparaffinized, rehydrated and retrieved in citrate buffer in microwave for 20 min. Sections were rinsed in PBS and incubated with peroxidase blocking solution (0.3% H_2_O_2_) for 10 min then rinsed with PBS again before incubation with 3 % normal horse serum blocking solution for 30 min then incubated with primary antibody mouse anti-GFAP (1:5,000), overnight. Brain sections were rinsed in PBS and incubated with horse radish peroxidase conjugated anti-mouse IgG complex (DakoCytomation) for 30 min and rinsed in PBS again before incubation with DAB peroxidase substrate solution for 5-10 min then rinsed in running tab water, dehydrated and covered. Three coronal sections were selected for image analysis. Regions of interest were delineated manually at magnification X100. The selected areas of dorsal hippocampus for astroglia analysis were stratum radiatum, and stratum oriens of CA1 and CA3, as well as inner and outer molecular layers and hilus area of DG. The percentage surface area of GFAP-positive astroglia was quantified in white matter area (WM); corpus callosum (CC), internal capsule (IC) and optic tract (OpT). Measurements were carried out in both sides of the hippocampus and WM areas (Farkas et al., 2005 ▶; Farkas et al., 2006 ▶).

**Statistical analysis**

Learning ability determined by escape latency (the time taken to find the platform) was analyzed by repeated measures analysis of variance (ANOVA) followed by Fisher’s *post hoc* test. Memory capacity determined by the time spent in the target arm (%), the percentage of dead cell and percent area of astroglia were analyzed by one-way ANOVA followed by Fisher’s *post hoc* test. The statistical significance was accepted if p< 0.05.

## Results

**Sensorimotor and cognitive assessments following 2 months of CCH (Set I)**

**Figure. 1:**
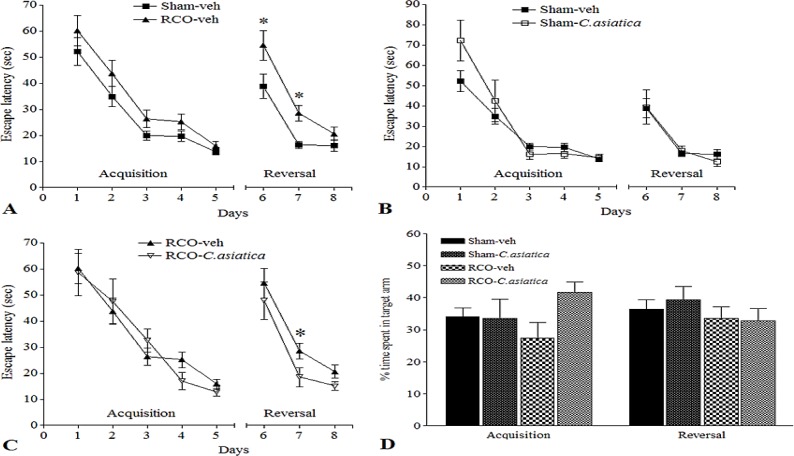
Spatial learning and memory following 2 months of CCH (n = 8 in each group). (A) The spatial learning deficit was found in RCO-veh rats on days 6 and 7 compared to Sham-veh rats; * p<0.05. (B)* C. asiatica* extract had no effect on Sham-*C. asiatica* compared to Sham-veh rats. (C) The learning flexibility was improved in RCO rats that received *C. asiatica* extract 20 mg/kg (RCO-*C. asiatica*), significant differences were found on day 7 (reversal phase) compared to RCO-veh; *p<0.05. (D) No significant difference was found in spatial memory in both acquisition and reversal phases

Assessment of the visibility and swimming ability on the pre-training day (cognitive test day 0) with visible platform revealed that sensorimotor ability was preserved in all rats. There was no significant difference in swimming speed among groups (Sham-veh 22.28±1.55 cm/sec, Sham-*C. asiatica* 24.83±6.25 cm/sec, RCO-veh 21.07±0.45 cm/sec and RCO-*C. asiatica* 28.71±3.77 cm/sec). The learning ability of RCO-veh and Sham-veh groups was not significantly different in acquisition phase but in the reversal phase, RCO-veh rats showed significant learning flexibility deficit (cognitive test day 6, p=0.0260 and day 7, p=0.0002, respectively). The percentage of time spent in the target arm by RCO-veh and the Sham-veh rats was not significantly different. The results imply that mild CCH induced by RCO induced learning flexibility deficit but did not affect learning ability and memory capacity (Figure 1A). Oral administration of *C. asiatica* extract 20 mg/kg for 14 consecutive days had no effect on learning ability in the acquisition phase of the RCO rats. The performance in the reversal phase was significantly improved (on day 7, p=0.0071) but spatial memory was not significantly different (Figure 1D). The results suggested that *C. asiatica* improves learning flexibility deficit that was induced by 2 months of CCH.

**The percentage of dead neurons in the dorsal hippocampus following 2 months of CCH**

**Figure. 2:**
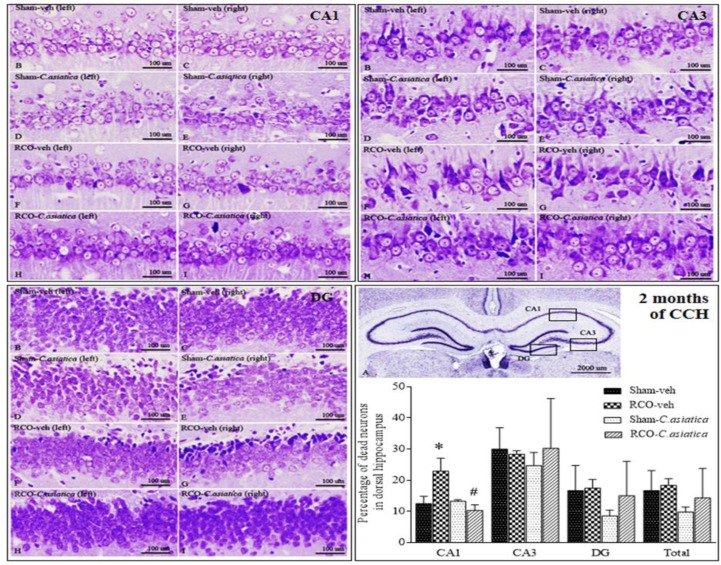
Representative photomicrographs of dorsal hippocampus of Sham-veh, Sham-*C. asiatica*, RCO-veh and RCO-*C. asiatica* rats, following 2 months of CCH. Panel A shows whole area of the dorsal hippocampus which consisted of CA1, CA3 and DG at 12.5X magnification. Panel B – I showed CA1, CA3 and DG areas at 200X magnification. Bar chart shows the percentage of dead neurons in the dorsal hippocampus (n=4 in each group). * p<0.05 compared to Sham-veh rats and ^# ^p<0.05 compared to RCO-veh rats

Results revealed that the percentage of dead neurons in the RCO-veh group was significantly higher than that of the Sham-veh group in CA1 area of the dorsal hippocampus (p<0.001). The percentage of dead neurons in the dorsal hippocampus of the Sham and the Sham-veh rats were not significantly different. *C. asiatica* treatment had no effect on the Sham rats. Whereas *C. asiatica* treatment, in the RCO group, significantly reduced the percentage of dead cell in the CA1. The percentage of dead neurons in the RCO-*C. asiatica* group was significantly lower than that of the RCO-veh rats (p<0.001, Figure 2). The results suggested that *C. asiatica* extract can prevent neuronal death in the CA1 hippocampus of CCH rats.

**Astroglia expression following 2 months of CCH**

**Figure. 3:**
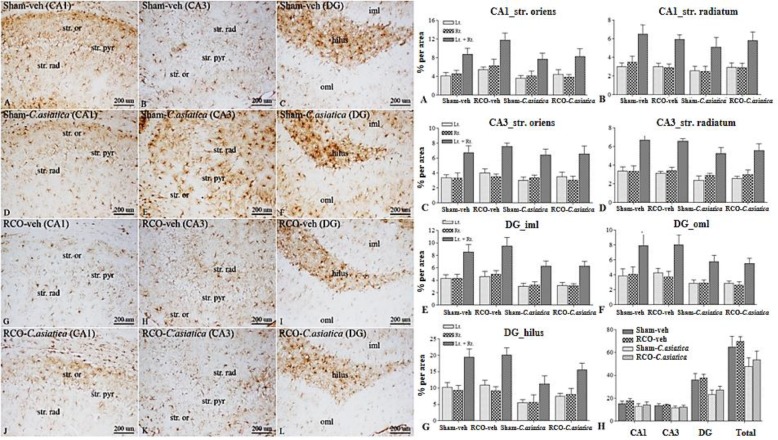
Representative photomicrographs of GFAP immunoreactivity in hippocampal area of interest, following 2 months of CCH (100X); str. or=stratum oriens, str. rad=stratum radiatum, str. pyr = stratum pyramidale, iml=inner molecular layer, oml = outer molecular. Panel A, D, G and J show GFAP immunoreactivity in CA1 area. Panel B, E, H and K show GFAP immunoreactivity in CA3 area. Panel C, F, I and L show GFAP immunoreactivity in DG area. Bar chart shows GFAP immunoreactivity (% area) in hippocampal area of interests (n = 4 in each group). Panel A and B represent CA1 stratum oriens and stratum radiatum, respectively. Panel C and D represent CA3 stratum oriens and stratum radiatum, respectively. Panel E, F and G represent DG inner molecular, outer molecular and hilus, respectively. Panel H represents GFAP immunoreactivity in all areas of hippocampus

**Figure. 4:**
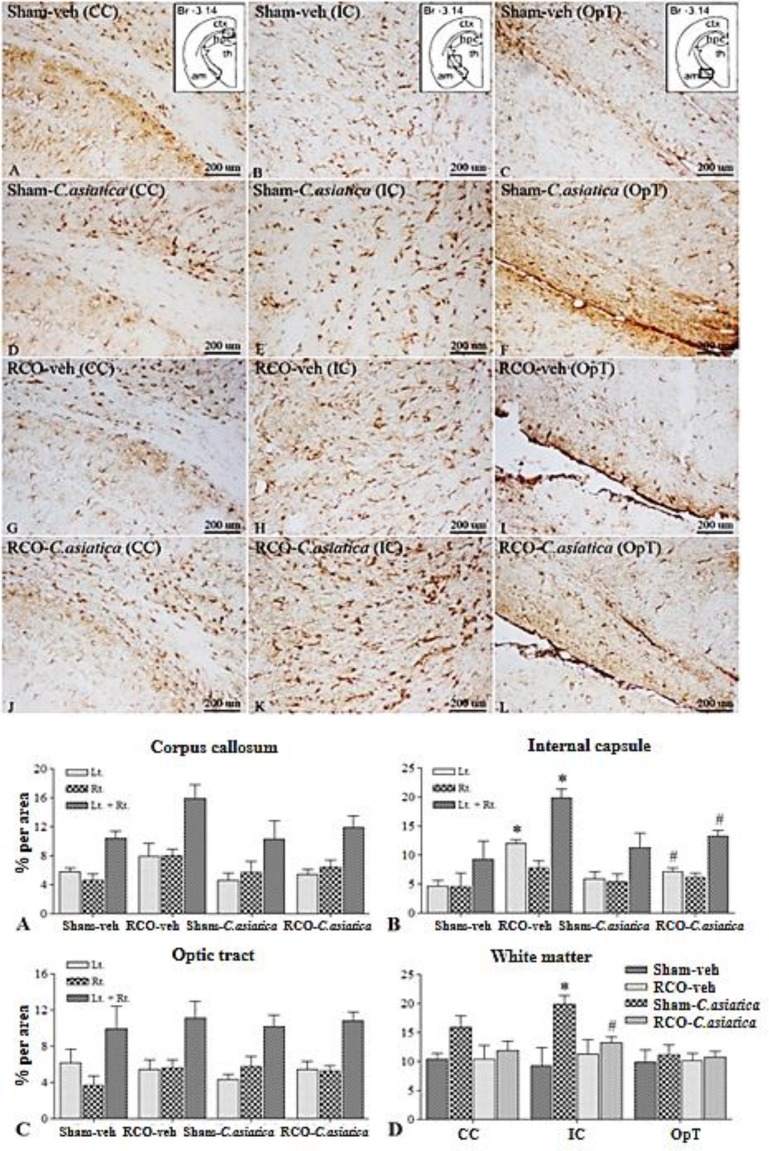
Representative photomicrographs of GFAP immunoreactivity in white matter area of interest namely, corpus callosum (CC), internal capsule (IC) and optic tract (OpT), following 2 months of CCH (100X magnification). Br = Bregma, ctx = cortex, hpc = hippocampus, th = thalamus and am = amygdala. Panel A, D, G and J show GFAP immunoreactivity in CC. Panel B, E, H and K show GFAP immunoreactivity in IC. Panel C, F, I and L show GFAP immunoreactivity in OpT. Bar chart shows GFAP immunoreactivity (% area) in white matter areas (n=4 in each group). * p<0.05 compared to Sham-veh and # p<0.05 compared to RCO-veh rats

Astroglia expression in dorsal hippocampus of RCO-veh was not significantly different in all areas of interest when compared to Sham-veh rats. Treatment with *C. asiatica* extract produced no significant effect on astroglia expression in the dorsal hippocampus as well (Figure 3). Astroglia expression in WM areas such as CC and OpT of the RCO-veh, was not significantly different from that of the Sham-veh rats. Significant differences between these two groups were found only in the IC (p=0.0032). Regarding astroglia expression in the WM, *C. asiatica* extract treatment did not induce significant differences when comparing Sham-veh rats and Sham-*C. asiatica* rats. Astroglia expression in IC of the RCO-*C. asiatica* rats was significantly reduced when compared to RCO-veh rats (p<0.05, Figure 4).

**Sensorimotor and cognitive assessments follwing 12 months of CCH (Set II)**

**Figure. 5:**
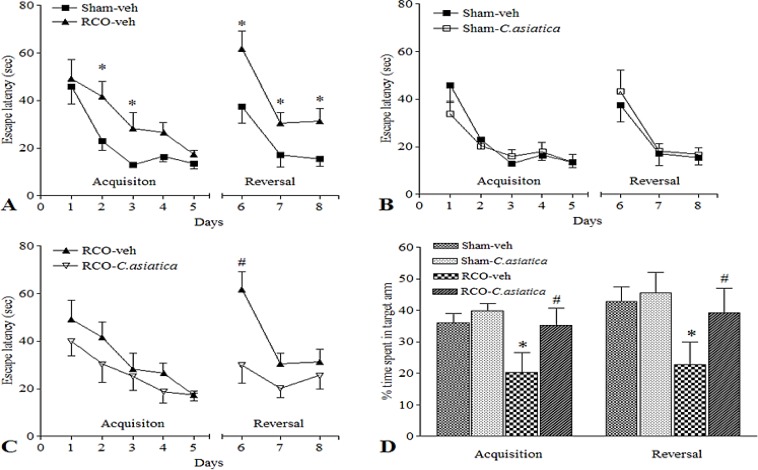
Spatial learning and memory in the RAWM test following 12 months of CCH. Sham-veh (n=8), Sham-*C. asiatica* (n=7), RCO-veh (n=8) and RCO-*C. asiatica* rats (n=8). * p<0.05 compared to Sham-veh rats and ^# ^p<0.05 compared to RCO-veh rats

Pre-training day cue test represented that the sensorimotor ability was preserved in all rats. There was no difference in the swimming speed among the groups (Sham-veh 24.68±2.84 cm/sec, Sham-*C. asiatica* 30.75±2.38 cm/sec, RCO-veh 28.03±2.55 cm/sec and RCO-*C. asiatica* 23.88±1.36 cm/sec). Spatial learning and memory after 12 months of RCO, showed significant differences among groups. Comparison between Sham-veh and RCO-veh showed significant differences in the escape latency on day 2 (p<0.05), day 3 (p<0.05), day 6 (p<0.05), day 7 (p<0.05) and day 8 (p<0.05). These data represented that CCH that was induced by RCO for 12 months, caused significant deficit in spatial learning. On days 6-8, considered the reversal phase, a clear impairment of learning flexibility was observed. Spatial memory capacity showed a similar trend as those of spatial learning and flexibility that were significantly impaired in RCO-veh rats. The percentage of time spent in the target arms by RCO-veh rats both in acquisition (p<0.001) and reversal probe (p<0.001) was significantly lower than that of the Sham-veh rats. These data suggested that CCH that was induced by RCO for 12 months, induces significant deficit in spatial learning, spatial memory and learning flexibility in RCO-veh rats (Figure 5).

The spatial learning and memory after *C. asiatica* treatment in Sham-*C. asiatica* showed no difference when compared to untreated rats (Sham-veh). The RCO treated with *C. asiatica* extract (RCO-*C. asiatica*) showed significant improvement of learning flexibility and memory compared to the untreated group (RCO-veh). Significant difference between these two groups were found on day 6, for learning (p<0.01) and for spatial memory (acquisition probe, p<0.001 and (reversal probe, p<0.001). The data suggested that *C. asiatica* extract treatment improves learning flexibility and spatial memory in RCO rats.

**The percentage of dead neurons in the dorsal hippocampus at 12 months of CCH**

**Figure. 6:**
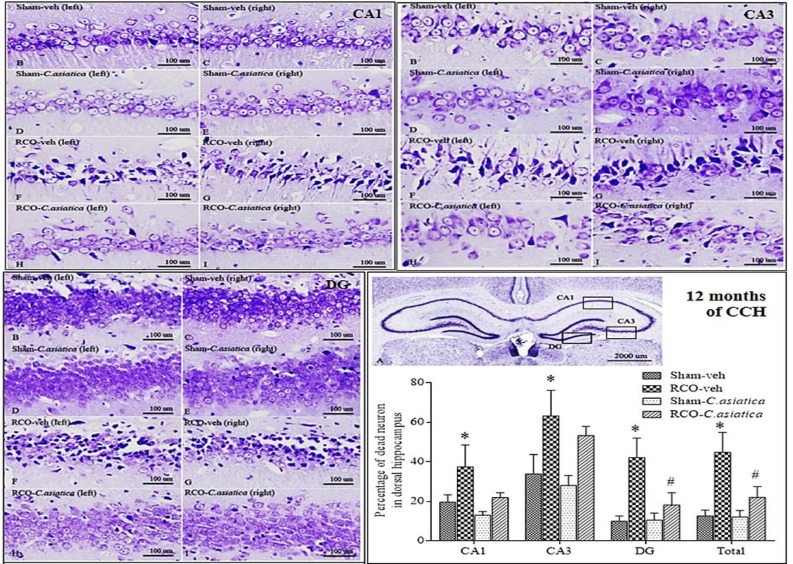
Representative photomicrographs of dorsal hippocampus of Sham-veh, Sham-*C. asiatica*, RCO-veh and RCO-*C. asiatica* rats, following 12 months of CCH. Panel A shows whole area of the dorsal hippocampus which consisted of CA1, CA3 and DG (12.5X magnification). Panel B – I shows CA1, CA3 and DG areas (200X magnification). Bar chart shows the percentage of dead neurons in the dorsal hippocampus (n=4 in each group). * p<0.05 compared to Sham-veh rats and ^#^ p<0.05 compared to RCO-veh rats

The percentage of dead neurons in the dorsal hippocampus of the RCO-veh rats was significantly higher than the Sham-veh rats in CA1, CA3, DG and total area of the dorsal hippocampus (p<0.01 for CA1;p<0.05 for CA3; p<0.01 for DG and p<0.01 for total area of the dorsal hippocampus). These data suggested that RCO for 12 months induces significant neuronal death in the dorsal hippocampus. The RCO rats which were given *C. asiatica* extract showed significant reduction of the percentage of dead neurons compared to the untreated rats (RCO-veh) only in the DG (p<0.001), and total area of the dorsal hippocampus (p<0.05, Figure 6). 

**Figure. 7:**
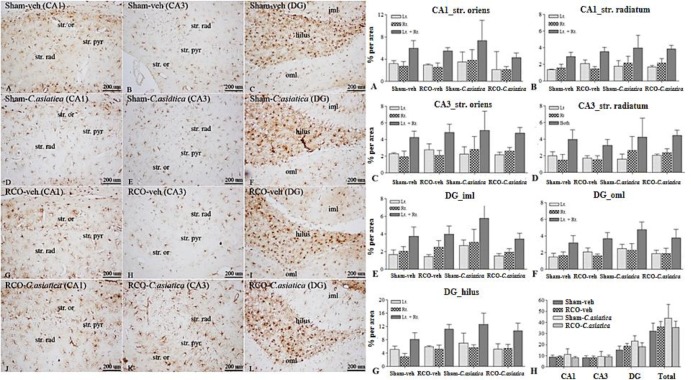
Representative photomicrographs of GFAP immunoreactivity in hippocampal area of interest, 12 months of CCH (100X magnification). str. or=stratum oriens, str. rad=stratum radiatum, str. pyr=stratum pyramidale, iml=inner molecular layer, oml=outer molecular. Panel A, D, G and J show GFAP immunoreactivity in CA1 area. Panel B, E, H and K show GFAP immunoreactivity in CA3 area. Panel C, F, I and L show GFAP immunoreactivity in DG area. Bar chart shows GFAP immunoreactivity (% area) in hippocampal area of interests (n=4 in each group). Panel A and B represent CA1 stratum oriens and stratum radiatum, respectively. Panel C and D represent CA3 stratum oriens and stratum radiatum, respectively. Panel E, F and G represent DG inner molecular, outer molecular and hilus, respectively. Panel H represents GFAP immunoreactivity in all areas of hippocampus

**Astroglia expression following 12 months of CCH**

**Figure. 8:**
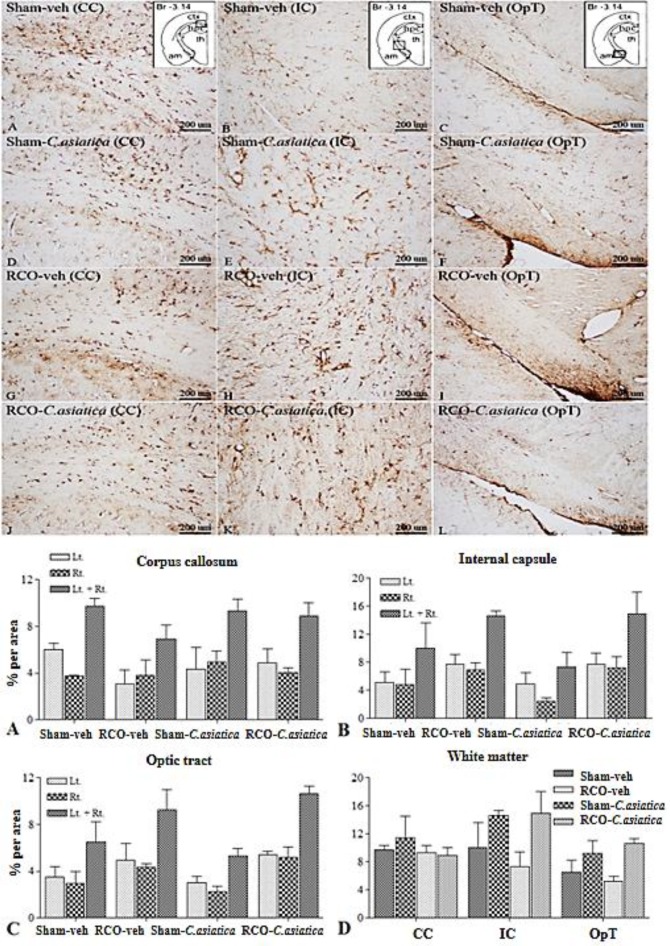
Representative photomicrographs of GFAP immunoreactivity in white matter area of interest namely, corpus callosum (CC), internal capsule (IC) and optic tract (OpT), following 12 months of CCH, (100X magnification). Br=Bregma, ctx=cortex, hpc=hippocampus, th=thalamus and am=amygdala. Panel A, D, G and J show GFAP immunoreactivity in CC. Panel B, E, H and K show GFAP immunoreactivity in IC. Panel C, F, I and L show GFAP immunoreactivity in OpT. Bar chart shows GFAP immunoreactivity (% area) in white matter areas, Sham-veh (n=3), Sham-*C. asiatica *(n=4), RCO-veh (n=3) and RCO-*C. asiatica *rats (n=3).

Astroglia expression in dorsal hippocampus of 12 months of CCH (RCO-veh) were not different in all areas of interest when compared to Sham-veh rats. Treatment with *C. asiatica* extract produced no effect on astroglia expression in dorsal hippocampus as well (Figure 7). Astroglia expression in WM areas such as CC, IC and OpT were not different when comparing RCO-veh and Sham-veh rats and was comparable to the* C. asiatica* extract treatment groups (Figure 8).

## Discussion

The present study investigated the effect of ECa-233 standardized *C. asiatica* extract on cognitive deficits and neuronal death in short-term and long-term CCH induced by permanent RCO in rats. We found that *C. asiatica* extract 20 mg/kg significantly improves learning flexibility and reduces the number of dead neurons in CA1 associated with reduction of glia activation in IC at 2 months of RCO. The efficacy of *C. asiatica* extract that was administered after long-lasting mild reduction in CBF was also depicted. We found that 12 months of RCO induces significant deficit in spatial learning, memory and learning flexibility. These cognitive deficits were associated with significant neuronal damage in the dorsal hippocampus but no changes in astroglia expression in both hippocampus and WM areas. Standardized *C. asiatica* extract treatment significantly improved spatial memory and learning flexibility in CCH rats. Late treatment with standardized *C. asiatica* extract was able to rescue hippocampal damage only in DG area of CCH rats. Our findings indicated the therapeutic potential of ECa-233 standardized *C. asiatica* extract either given immediately after ischemic insult or administered after long-lasting ischemic insult in a CCH rat model.

Two months of CCH significantly induced deficits in learning flexibility (Thong-asa et al., 2013 ▶). Switch platform protocol in the reversal phase by changing platform position in the acquisition phase to new position, challenges the rats to inhibit swimming to the old location and to swim to a new position indicating a form of behavioral flexibility (McDonald et al., 2008 ▶). The dorsomedial striatum and intrinsic acetylcholine actions play a pivotal role in platform-switch response learning by facilitating learning of a new response and inhibiting the old response (Ragozzino, 2003 ▶). There are several neural structures that are implicated in behavioral flexibility including the medial prefrontal cortex, dorsomedial striatum and hippocampus (Ragozzino, 2003 ▶; Ragozzino et al., 1999 ▶). Impairment of behavioral flexibility may be due to damage to those brain areas that are responsible for this function or disconnectivity among these brain areas. Two months of RCO induced significant damages only in the CA1 area which was associated with the deficit in learning flexibility in the present study. However, hippocampal damage alone may not account for the deficit in learning flexibility. Once the platform location was changed, it required other brain areas such as medial prefrontal cortex and dorsomedial striatum to take this responsibility (McDonald et al., 2008 ▶). The hippocampus only plays a key role in navigation function in learning performance in the acquisition trial and the learning performance in reversal trial might be relevant to the medial prefrontal and dorsomedial striatum. Damages in CA1 hippocampus might not fully explain this learning flexibility deficit alone. It was reported that 1 month of RCO in mice resulted in WM damage in the corpus callosum as reflected by WM rarefaction and decreased fiber density (Yoshizaki et al., 2008 ▶). White matter damage may play a key role in disconnection of the fronto-subcortical circuit. Our findings revealed significant increases in glia activation in the IC. The IC links the cerebral cortex to subcortical structures. It contains the anterior thalamic peduncle, which connects the medial and anterior thalamic nuclei with the prefrontal cortex which is involved in two functionally important circuits namely, the medial limbic circuit and the basolateral limbic circuit. Abnormalities in the IC could therefore interfere with the flexibility in spatial learning (Wobrock et al., 2008 ▶). The precise damage of this structure was not investigated in the present study but possibly it was related to anatomical connectivity. Astroglia are involved in most of integrated functions of the central nervous system that are not only necessary for the normally working brain but are also critically involved in many pathological conditions, including stroke. They may contribute to damage by propagating spreading depression or by sending pro-apoptotic signals to healthy tissue via gap junction channels. Astroglia also inhibit regeneration by participating in formation of the glial scar. They are also important in neuronal antioxidant defense and secrete growth factors, which probably provide neuroprotection in the acute phase (Anderson et al., 2003 ▶; Chen and Swanson, 2003 ▶). As major reservoirs of antioxidants (such as GSH and ascorbate) for neurons, astroglia provide defensive mechanism against oxidative stress in the brain. In an ischemic situation, ROS increase and cause neuronal damage. As relevant to the present study, ROS might increase at the period of 2 months after RCO in the IC and further activate astroglia for defensive mechanism.

No significant learning impairment was seen in acquisition trial at 2 month of RCO. It has been shown that normal acquisition of a spatial memory task in rats depends on the volume and location of hippocampal tissue. Only a small transverse block (minislab) of the hippocampus (almost 26% of the total) could support spatial learning in a water maze (Moser et al., 1995 ▶). Therefore, little damage to CA1 hippocampus after RCO may not reach the critical volume required for learning deficits.

Orally administered ECa-233 standardized* C. asiatica* extract 20 mg/kg for 14 consecutive days significantly improved learning flexibility and reduced the number of dead neurons in CA1 after 2 months of RCO. *C. asiatica* extract treatment exerted these beneficial effects only in CCH rats but not in normal rats (Sham-*C. asiatica*). This might show that *C. asiatica* extract exerts its neuroprotective effects only under pathological condition. Energy crisis in the CCH state did not reach the level needed to kill the neurons, but only caused neuronal hypofunction, dysfunction and eventually cell dead. The loss of ATP could result in dysfunction of energy-dependent ionic pump, leading to depolarization, excessive release of glutamate, and generation of ROS which are lethal to neurons at high concentrations. Numerous studies demonstrated that* C. asiatica* protects cells under energy crisis by protecting them from glutamate-induced excitotoxicity and activation intrinsic antioxidant activity. It has been shown that *C. asiatica* treatment significantly reduces oxidative stress such as lipid peroxidation and protein carbonyl and activates antioxidant enzymes e.g. GSH, GSHPx and catalase in both normal adult, normal aging and AD rat model (Hussin et al., 2007 ▶; Jayashree et al., 2003 ▶; Lee et al., 2000 ▶; Veerendra Kumar and Gupta, 2002 ▶, 2003; Zainol et al., 2003 ▶). *C. asiatica* that was used in the present study contained 85% of total triterpenoids which were 53% of madecassoside and 32% of asiaticoside. A previous study using *C. asiatica* components reported improvement of learning and memory deficit induced by bilateral common arteries occlusion in both MWM and step-down tests together with amelioration of MDA level (Wanasuntronwong et al., 2012 ▶). This indicates that the improvement of cognitive function might be associated with the plant’s anti-oxidant properties. Furthermore, *C. asiatica* also had nootropic activity in enhancing cognitive ability via acetylcholine activity. *C. asiatica* extract-treated rats showed improvement in cognitive ability in passive avoidance and elevated plus-maze paradigms (Veerendra Kumar and Gupta, 2002 ▶, 2003), Morris water maze and step-down paradigms, radial arm maze (RAM) and hole board paradigm (Rao et al., 2005 ▶). As a result of brain energy metabolism alteration in CCH, glia activation was prominently found in IC in short-term CCH in the present study and *C. asiatica* treatment reversed it. This was similar to a previous report by Raghavendra et al. (2009) ▶ in which, *C. asiatica* treatment reversed gliosis, perivascular oedema, mononuclear cell infiltrates and glia activation in CCH rats together with alleviation of cognitive and histopathological changes (Raghavendra et al., 2009 ▶). 

The present study is the first report to demonstrate the long-lasting effect of RCO on the unexperienced spatial performance of the RCO rats. It was observed that 12 months of RCO significantly induces deficit in both spatial learning and memory in the acquisition phase as well as learning flexibility and memory in the reversal phase. These deficits were found along with significant CA1 hippocampal damage; but, no changes in hippocampal and WM astroglias were observed. Long-lasting reduction of CBF was found to exacerbate spatial reference and working memory in the RAM. Sopala et al. (2001 ▶) demonstrated that advanced age alone exerted significant cognitive deficit in old rats (16 months old). The age-induced cognitive deficit was deteriorated when combined with long-lasting reduction of CBF. The long-lasting reduction of CBF combined with aging enhances cognitive impairment of aged individuals (Sopala and Danysz, 2001 ▶). Also, age-related memory disorders was demonstrated to be accompanied by structural changes in the hippocampus in 12-month old rats (Kadar et al., 1990 ▶). It is well known that aging causes structural, biochemical and functional changes in the brain. Aging alone only has a modest effect on neuronal loss and only affects certain populations of neurons. Besides loss of neurons, dendritic and axonal arborization sizes were reduced. These changes were induced by free radicals resulting from a high rate of oxidative metabolism in neurons, glycation and dysregulation of intracellular calcium homeostasis (Esiri, 2007 ▶; Jernigan et al., 2001 ▶).

Spatial learning and memory deficit in long-term occlusion of the right common carotid artery in the present study resulting from both CCH and aging which converge to exacerbate the effect of aging, increased oxidative stress and free radical formation. Kadar and his colleagues (1990) reported significant memory impairment at the age of 12 months in RAM which did not further decline when assessed again at 17 months of age. Morphometric analysis also revealed a decrease in the area of cells within the hippocampus. The cells reduction in the CA3 subfield suggests age-related behavioral deficits and shows that the hippocampal CA3 region plays a major role in the age-dependent cognitive decline (Kadar et al., 1990 ▶). The present study found significant damages in CA3, DG and dorsal hippocampus in RCO-veh rats, but it could not completely postulate about how much of aging participated in this neuronal damage, spatial learning and memory deficits. 

*C. asiatica* extract exerts beneficial effects on cognitive functions and mood (Gadahad et al., 2008 ▶; Rao et al., 2005 ▶; Sarma et al., 1996 ▶; Veerendra Kumar and Gupta, 2002 ▶, 2003; Wattanathorn et al., 2008 ▶; WHO, 1999 ▶). The effect of *C. asiatica* extract on neuronal protection from the effect of long-lasting reduction of CBF combined with aging might be due to its antioxidant properties. As it was proposed by Farkas et al. (2007) ▶ that CCH-induced neuronal death is mainly mediated via apoptotic activation as the result of suboptimal levels of ATP, increased ROS and inhibition of anti-oxidation processes as a typical of ischemic brain injury (Farkas et al., 2007 ▶). Permanent ischemic/oligemic conditions are enough serious to sustain continuous oxidative stress which induces sustained progressive neuronal damage (Aytac et al., 2006 ▶). The oxidative stress activation leading to brain damage and alteration of brain functions, also represented in normal aging brain (Esiri, 2007 ▶). Hence, the damages to the hippocampal neurons seen in the present study could come from both conditions. It was not surprising that *C. asiatica* extract treatment improved both functional deficit and structural damage. *C. asiatica *extract treatment improved not only hippocampal function (spatial memory), but also other brain functions related to the hippocampus such as behavioral flexibility. The area that might be damaged by both aging and CCH was WM which connects the fronto-hippocampal-striatal circuit. Since *C. asiatica* treatment significantly reduced DG hippocampal damage but not CA1 or CA3, neurogenesis processes might be involved. There is another report suggesting that *C. asiatica* has a promoting effect on neurogenesis through induction of differentiation of neural precursor cells which can be found in the DG of adult brains (Haeun et al., 2015 ▶). This might explain why 12 months of CCH with *C. asiatica* extract treatment caused significant reduction in neuronal damage only in DG rather than CA1 or CA3 of the dorsal hippocampus in the present study.

Our findings indicated the therapeutic potential of standardized *C. asiatica* extract (ECa-233) given either immediately after ischemic insult or one year after ischemic insult to CCH rats. CCH after 2 months of permanent RCO induced learning flexibility deficits along with CA1 neuronal damage and IC astroglia activation. Long-lasting CBF reduction for 12 months induced spatial learning, memory and flexibility deficits associated with progressive damages in dorsal hippocampus. Treatment with ECa-233 improved learning flexibility deficit following 2 and 12 months of CCH. ECa-233 ameliorated neuronal damage in the dorsal hippocampus after 2 months of CCH when given immediately after CCH onset. Administration of ECa-233 after long-lasting CBF reduction as 12 months of CCH improved memory and learning flexibility deficits and it was accompanied by reductions in neuronal damage in the DG.
